# Classification-Based Prediction of Effective Connectivity Between Timeseries With a Realistic Cortical Network Model

**DOI:** 10.3389/fncom.2018.00038

**Published:** 2018-06-05

**Authors:** Emanuele Olivetti, Danilo Benozzo, Jan Bím, Stefano Panzeri, Paolo Avesani

**Affiliations:** ^1^NeuroInformatics Laboratory (NILab), Bruno Kessler Foundation, Trento, Italy; ^2^Center for Mind and Brain Sciences (CIMeC), University of Trento, Trento, Italy; ^3^Information Engineering and Computer Science Department (DISI), University of Trento, Trento, Italy; ^4^Neural Computation Laboratory, Istituto Italiano di Tecnologia, Rovereto, Italy

**Keywords:** causality, classification and prediction, cortical network model, neural networks, connectivity, effective connectivity, timeseries analysis

## Abstract

Effective connectivity measures the pattern of causal interactions between brain regions. Traditionally, these patterns of causality are inferred from brain recordings using either non-parametric, i.e., model-free, or parametric, i.e., model-based, approaches. The latter approaches, when based on biophysically plausible models, have the advantage that they may facilitate the interpretation of causality in terms of underlying neural mechanisms. Recent biophysically plausible neural network models of recurrent microcircuits have shown the ability to reproduce well the characteristics of real neural activity and can be applied to model interacting cortical circuits. Unfortunately, however, it is challenging to invert these models in order to estimate effective connectivity from observed data. Here, we propose to use a classification-based method to approximate the result of such complex model inversion. The classifier predicts the pattern of causal interactions given a multivariate timeseries as input. The classifier is trained on a large number of pairs of multivariate timeseries and the respective pattern of causal interactions, which are generated by simulation from the neural network model. In simulated experiments, we show that the proposed method is much more accurate in detecting the causal structure of timeseries than current best practice methods. Additionally, we present further results to characterize the validity of the neural network model and the ability of the classifier to adapt to the generative model of the data.

## 1. Introduction

To understand how the human brain works, it is fundamental to study the interactions among its regions and not just their individual behavior (see Bullmore and Sporns, 2009). The pattern of *causal* interactions between the temporal behavior of brain regions is called *effective connectivity* (see Sporns, 2007; Friston, 2011). Inferring such pattern from observations, e.g., from functional neuroimaging data, is a challenging task. Traditionally, the literature addresses this problem either with non-parametric, i.e., model-free, or parametric, i.e., model-based, methods (see Chicharro, 2014). In particular, model-based methods define a generative model of the dynamics of the neural system and then estimate the parameters of the model, e.g., the pattern of causal interactions, from the data, by inverting the model. The most well-known among such models are the multivariate autoregressive (MAR) model (Granger, 1969) and the dynamic causal model (DCM) for functional MRI data (Friston et al., 2003).

Parametric methods are very popular when studying effective connectivity in the brain. When the model used to parametrize neural activity and their dependencies is biophysically plausible, these models can be used not only to infer causal relationships between neural circuits, but also to infer the mechanisms leading to it. Nevertheless, common models are either simplistic with respect to physiology (for example the MAR model is not based on physiological mechanisms), or specific for certain neuroimaging modality (DCM for fMRI), or they ignore, for simplicity, some important aspects of neural dynamics. For example, standard version of DCMs consider only the mean field dynamics of these models and ignore the rich structure of the neural dynamics that is not captured by mean field approximations.

Recent progress in neural network modeling has made it possible to generate models of recurrent microcircuits that have biophysical and anatomical properties very similar to those of real cortical circuits (see Brunel and Wang, 2003; Borisyuk and Kazanovich, 2006; Mazzoni et al., 2008, 2010, 2015; Kirst et al., 2016; Palmigiano et al., 2017). Moreover, when used to simulate dynamical systems, these models generate statistics very close to that of recorded cortical activity and of neural communication (see Belitski et al., 2008). Besserve et al. (2015) including realistic rapid fluctuations, such as gamma oscillations, that are not well-captured by simplified solutions of neural dynamics, such as the mean field. In principle, these realistic models could be used for studying effective connectivity from observations. However, unfortunately, estimating effective connectivity from observed data, i.e., inverting these complex models, is a complex task with no clear solutions available.

In recent years, the machine learning community has proposed to recast the problem of causal inference as a statistical learning theory problem (see Schölkopf et al., 2013; Lopez-Paz et al., 2015a,b; Mooij et al., 2016). The underlying idea is to use machine learning algorithms on observed data, using a supervised learning paradigm. Different solutions have been devised, even though not targeting causality between multivariate timeseries and not addressing generative models of brain activity. Moreover, a limitation of these approaches is the lack of a large amount of data for training the algorithms, an issue typical of some domains of application, such as neuroscience.

In this work, we explore a possible new way to predict effective connectivity taking advantage of biophysically plausible cortical models. We first perform a novel extension of the neural network model of Mazzoni et al. (2008) where we add interactions between neural populations. We then propose a method to address the limitations of (i) inverting complex generative models to detect causality among timeseries and, jointly, (ii) of using the supervised learning framework when observations are in limited number. Notice that, here, we define causality as binary, i.e., given one multivariate timeseries composed of *M* timeseries, its pattern of causality is a *M*×*M* binary matrix where entry *ij* is 1 if timeseries *i* causes timeseries *j*, and 0 otherwise. Our idea is to use the generative model to simulate a large number of examples, where one example is a multivariate timeseries together with its pattern of causality. We use the resulting dataset of examples to train a classification algorithm that, given a new multivariate timeseries as input, predicts its pattern of causality. In this way, the proposed supervised method *approximates* the result of model inversion [cf. (i)] and, moreover, does not require any real observations to be trained but only simulated data [cf. (ii)]. Of course, a crucial desideratum of the proposed method is to provide *accurate* approximations, i.e., to accurately predict effective connectivity.

The method presented in this work builds on our previous work (see Benozzo et al., 2016, 2017), where we developed a conceptually similar paradigm applied to a simple MAR model, rather than to a complex biophysical model of neural interactions. There, we proposed a novel feature space to encode the multivariate timeseries and showed that effective connectivity could be predicted at even better rates than those of state-of-the-art solutions. Rather than using the MAR model, here we propose a novel neural network generative model, which extends the work of Mazzoni et al. (2008). By connecting several of the recurrent microcircuits, as shown in section 2.2, we can generate a simulated information flow among neural circuits that has realistic statistical properties and for which we know the ground truth of causal communication. Notice that, a method for estimating the pattern of causality expressed by this model, from observed data, is not available and it is not straightforward.

In order to set up a classification problem, here we adopt the feature space that we designed in Benozzo et al. (2016, 2017), which is based on the Granger principles of temporal precedence and predictability. With such feature space, we obtain a more convenient representation of the multivariate timeseries generated by the neural network model. Then, our main result is to show that the resulting classifier can accurately predict the pattern of causality of multivariate timeseries, even in the case of the proposed complex generative model based on neural networks, so demonstrating accurate approximate model inversion. As additional result, we also provide evidence that the neural network model proposed in section 2.2 implements the Granger principles, despite being vastly different from a MAR model. As a further result, we also compare the proposed supervised method with the Granger Causality Analysis (GCA, see Barnett and Seth, 2014) method, commonly used to detect causality among timeseries from neural recordings. We show that the proposed method vastly outperforms GCA.

In the remaining part of the article, we first describe the proposed neural network model derived from Mazzoni et al. (2008), that we extended by adding connections between microcircuits rather than studying single isolated microcircuits as in the original model (see section 2). Then, we briefly describe the proposed method for detecting the pattern of effective connectivity from observed timeseries, following Benozzo et al. (2016, 2017). In section 3, we report the experiments conducted in this work to support our claims. First, in section 3.1, we characterize the proposed neural network model, showing that it does generate timeseries according to the given pattern of causality. Then, in section 3.2, on data generated by the neural network model, we present the main result, where we show the clear superiority of the proposed supervised method with respect to GCA. Additionally, in order to show the flexibility of the supervised method in adapting to the generative model, we show the effect of training on MAR data vs. neural network data. In section 4, we discuss the experimental findings and their support to our claims.

## 2. Materials and methods

In this section, after describing the causal configurations adopted in this study, we explain in detail the novel generative model for the activity of brain regions, based on neural networks. There, we provide the details of the parameters to generate the large set of examples, which was used to train and test the proposed classification-based method for predicting causality. Each example is composed of a multivariate timeseries and its causal configuration matrix. The remaining part of the dataset was used to estimate the quality of predictions, both for the proposed method and for the Granger Causality Analysis (GCA, see Barnett and Seth, 2014) method (see section 3), for comparison. In the second part of this section, we briefly present the proposed classification-based method for predicting causality, following Benozzo et al. (2016, 2017). Before the second part, we define the multivariate autoregressive (MAR) model, that we use for generating a second dataset to further characterize the proposed method, and a traditional causality measure: the Geweke index, on which GCA is based.

### 2.1. Causality configurations

In this work, we consider the causal relationships between three timeseries. This is the minimum number to observe complex multivariate causal interactions. Even with such number of timeseries, the amount of possible causal configurations is remarkable. In principle, the number of distinct configurations that *M* timeseries can exhibit with their (binary) causal interactions is super-exponential: 2^*M*(*M*−1)^. A straightforward way to obtain this is to consider the *M*×*M* adjacency matrix *A* of the directed graph describing such interactions, where each off-diagonal element can assume a binary value: the element *a*_*ij*_ = 1 when timeseries *i* causes timeseries *j* and 0 otherwise.

In the literature of causality it is common to consider only a subset of all directed graphs when describing causal relationships: the directed acyclic graphs (DAGs) between labeled nodes. This restriction is inherent to many causality frameworks, e.g., Bayesian networks (Pearl, 2009), because the representation of probability distributions can leverage conditional independence relations (see Spirtes et al., 2001). In this way, the conditional independence properties that are inferred from data can be interpreted as causal properties (see Dawid, 2010).

When restricting to DAGs, the number *a*_*M*_ of possible causal configurations of *M* timeseries is still super-exponential (see Robinson, 1977)

(1) $$a_{M}=\sum_{k\;=\;1}^{M}{\left(-1\right)}^{k-1}\left(\begin{array}{c} M\\ k \end{array}\right)2^{k(M-k)}a_{M-k}.$$

**Figure 1:**
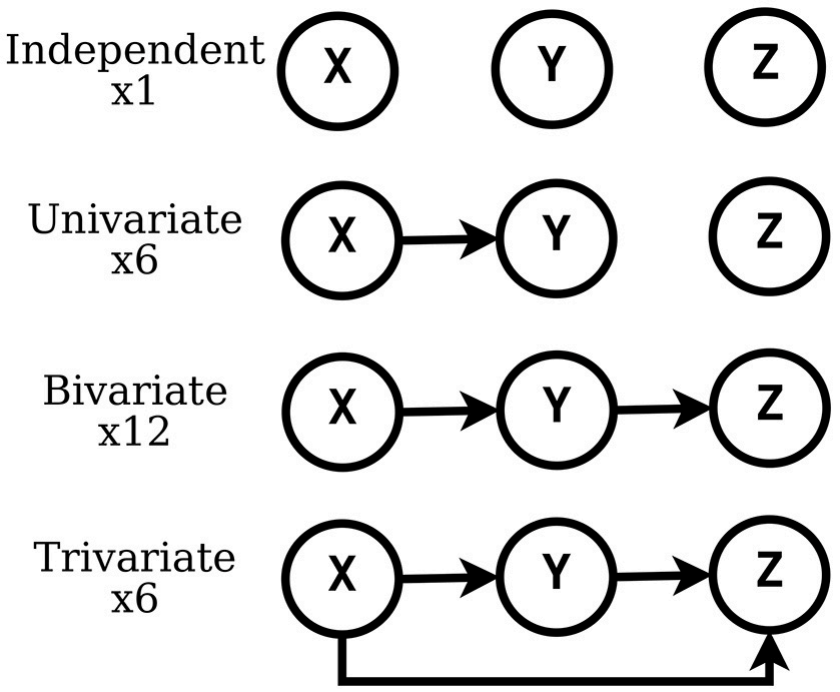
Possible DAGs exhibited among 3 labeled nodes.

In this work, we consider the causal interactions between three timeseries and, specifically, those expressed by DAGs, which amount to 25 configurations. In Figure 1, we list such configurations, grouped as independent, univariate, bivariate and trivariate.

### 2.2. Neural network (NN) model and dataset

In this subsection, we describe the biophysically plausible neural network model of causal communications between areas that we propose. We used such model to make inferences about brain connectivity from simultaneous measures of activity of multiple neural circuits. To develop this model, we took the model previously developed by Mazzoni et al. (2008) for describing the dynamics of individual neural circuits, and we generalized it to model multiple circuits connected to each other.

**Figure 2:**
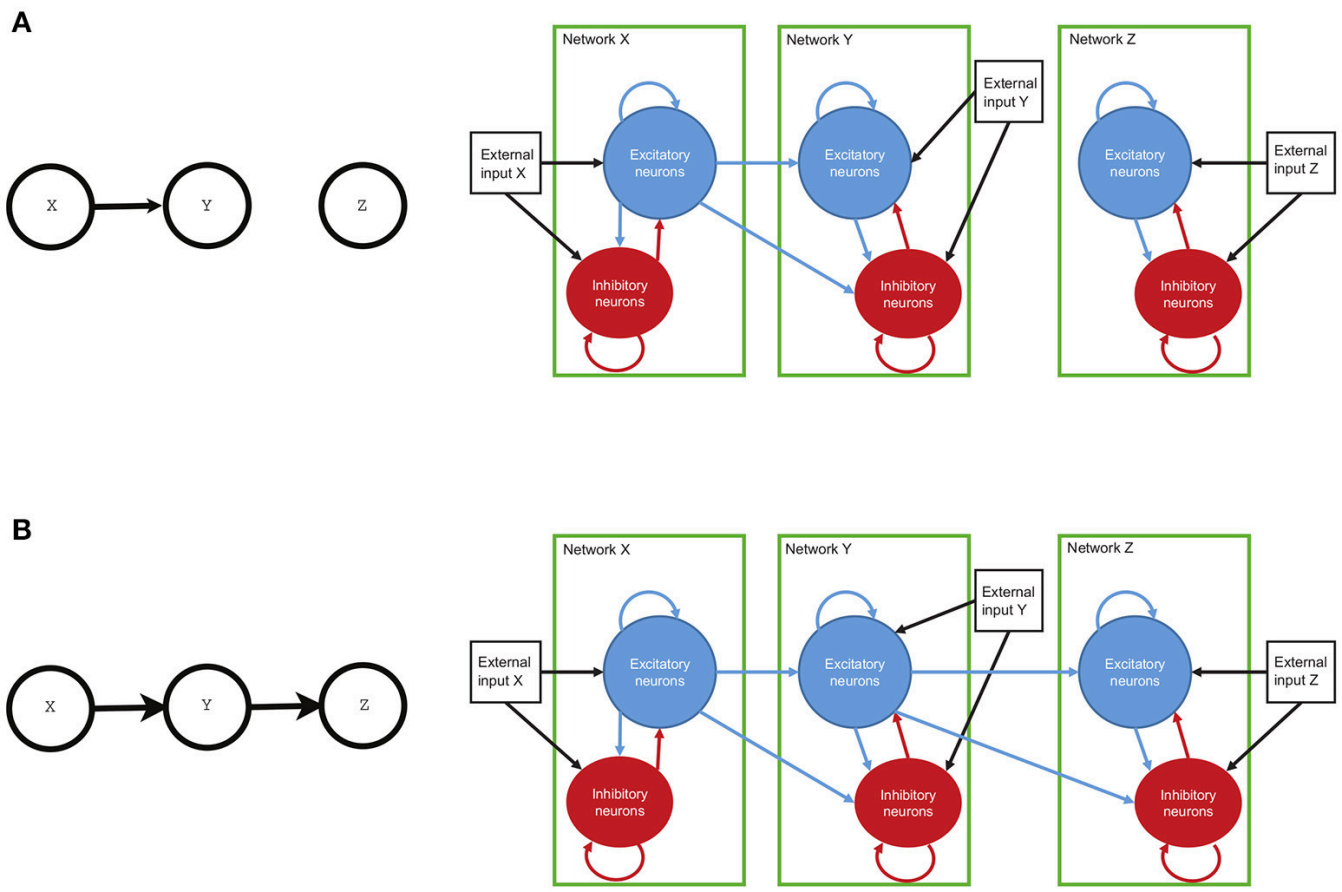
The connectivity of the network and the high-level structure of neural circuits. Both circuits of neurons, with each circuit made of interconnected excitatory and inhibitory neurons, have connections within the circuit and also with other circuits. Each neuron may also receive an external input, representing incoming information from neural circuits other than those modeled. In the first circuit (circuit X, right panels) the external input is a simulated spike train and is thought to represent input from the visual periphery (the Lateral Geniculate Nuclues, which in turn receives visual information from the eye). In other circuits (circuits Y and Z), this external input represents inputs from other possible cortical circuits non included in the model. Finally, if a connection between individual circuits is present (arrows), there are directed connections from the excitatory circuit of the sender to both excitatory and inhibitory neurons in the receiving network. **(A)** represents univariate connection from network **X** to network **Y**. **(B)** depicts bivariate connection between networks. The information flows from network **X** to network **Y** and from there to network **Z**.

The single circuit model of Mazzoni et al. (2008) was shown to produce simulated timeseries that are very similar to the statistics of physiological recordings, both at the level of Local Field Potentials (LFP). The LFP is a graded potential that measures the mass activity of a local neural circuit around the tip of the electrode (see Einevoll et al., 2013). In Mazzoni et al. (2008), the authors presented a model of a cortical network composed of leaky integrate and fire neurons and they showed that this model produces activity with characteristics very similar to that recorded from primary visual cortex, both during spontaneous activity and during naturalistic visual stimulation. For our purpose, here we built a new model made of three such models of individual neual circuits, **X**, **Y**, and **Z**, separately. Then, we connected the three circuits according to three different connectivity configurations (see Figures 1, 2). In this work, we study the causal interactions between these three circuits. Then, the high level graph of causal interactions comprises three nodes, each related to one circuit.

Each circuit is composed of N = 5,000 neurons. 80% of the neurons are excitatory and the remaining 20% are inhibitory (see Braitenberg and Schüz, 1991). Each individual circuit has randomly distributed connections. The within-circuit connection probability between any directed pair of cells is 0.2 (Sjöström et al., 2001; Holmgren et al., 2003). In case of an inter-network directed connection, there is also 0.2 probability of connection between any pair composed of any cell from the receiver network and an excitatory cell from the sender network (see Figure 2). Both pyramidal (excitatory) neurons and interneurons (inhibitory) are described by leaky integrate and fire (LIF) dynamics (Tuckwell, 1988). Each neuron *k* is described by its membrane potential *V*_*k*_ that evolves according to

(2) $$\tau_{m}\frac{dV_{k}}{dt}=-V_{k}+I_{Ak}-I_{Gk}$$

where τ_*m*_ is the membrane time constant (20 ms for excitatory neurons, 10 ms for inhibitory neurons (see McCormick et al., 1985), *I*_*Ak*_ are the (AMPAtype) excitatory synaptic currents received by neuron *k*, while *I*_*Gk*_ are the (GABA-type) inhibitory currents received by neuron *k*. Note that, in Equation (2), we have taken the resting potential to be equal to zero. When the membrane potential crosses the threshold *V*_*thr*_, i.e., 18 mV above resting potential, the neuron fires, causing the following consequences: (i) the neuron potential is reset at a value *V*_*res*_, i.e., 11 mV above resting potential, (ii) the neuron can not fire again for a refractory time τ_*rp*_, i.e., 2 ms for excitatory neurons and 1 ms for inhibitory neurons.

Synaptic currents are the linear sum of contributions induced by single pre-synaptic spikes, which are described by a difference of exponentials. They can be obtained using auxiliary variables *x*_*Ak*_, *x*_*Gk*_. AMPA and GABA-type currents of neuron *k* are described by

(3) $$\tau_{dA}\frac{dI_{Ak}}{dt}=-I_{Ak}+x_{Ak}$$

(4) $$\begin{array}{l} \tau_{rA}\frac{dx_{Ak}}{dt}=-x_{Ak}+\tau_{m}(J_{k-exc}\sum_{exc}\delta (t-t_{k-exc}-\tau_{L})+\\ \;+J_{k-int}\sum_{int}\delta (t-t_{k-int}-\tau_{L-int})\\ \;+J_{k-ext}\sum_{ext}\delta (t-t_{k-ext}-\tau_{L})J_{k-exc}) \end{array}$$

(5) $$\tau_{dG}\frac{dI_{Gk}}{dt}=-I_{Gk}+x_{Gk}$$

(6) $$\tau_{rG}\frac{dx_{Gk}}{dt}=-x_{Gk}+\tau_{m}\left(J_{k-inh}\sum_{inh}\delta (t-t_{k-inh}-\tau_{L})\right)$$

where *t*_*k*−*exc*/*inh*/*int*/*ext*_ is the time of the spikes received from excitatory neurons/inhibitory neurons/inter-network excitatory neurons (if a connection from another network is present) connected to neuron *k*, or from external inputs, as explained see below. τ_*dA*_ (τ_*dG*_) and τ_*rA*_ (τ_*rG*_) are respectively the decay and rise time of the AMPA-type (GABA-type) synaptic current. τ_*L*_ = 1 ms and τ_*L*−*int*_ = 3 ms are latencies of post-synaptic currents for intra- and inter-network connections respectively. *J*_*k*−*exc*/*inh*/*int*/*ext*_ is the efficacy of the connections from excitatory neurons/inhibitory neurons/inter-network excitatory neurons/external inputs on the circuit of neurons to which *k* belongs.

As noted above, each neuron is receiving an external excitatory synaptic input, see the last term in the r.h.s. of Equation (4). These synapses are activated by random Poisson spike trains, with a time varying rate which is identical for all neurons. This rate is given by

(7) $$\nu_{ext}(t)={\left[\nu_{signal}(t)+n(t)\right]}_{+}$$

where ν_*signal*_(*t*) represents the signal, and *n*(*t*) is the noise. [⋯ ]_+_ is a threshold-linear function, [*x*]_+_ = *x* if *x*>0 and 0 otherwise, to avoid negative rates which could arise due to the noise term. We use constant signal defined by

(8) $$\nu_{signal}(t)=\nu_{0}$$

where ν_0_ is a constant rate equal to 2 spikes/ms. The noise represented by *n*(*t*) in Equation (7) is generated according to an Ornstein-Uhlenbeck process.

The activity of each network was summarized by generation of simulated local field potential (LFP), as timeseries. To capture in a simple way the fact that pyramidal cells contribute the most to LFP generation, the LFPs are modeled as the sum of the absolute values of AMPA and GABA currents (|*I*_*A*_|+|*I*_*G*_|) on pyramidal cells in every time point of the simulation.

In previous studies, it was shown that this simple approximation of LFP, in terms of synaptic currents, is sufficiently accurate. It captures a large part of the variance, of the order of 95%, of complex LFPs generated by the sum of the dipoles generated in networks of model neurons with complex 3-dimensional structures and parameters closely matching those of real cortical neurons (see Mazzoni et al., 2015).

In all scenarios of Figure 1 we simulated three networks with the same set of parameters. However, their internal connections and external inputs were generated independently. All the parameter values were set in agreement with the original work of Mazzoni et al. (2008), with addition of synaptic efficacies for inter-network connections *J*_*k*−*int*_, that were equal for excitatory and inhibitory neurons. These values were drawn from a uniform distribution in the interval [0, 0.18], for every pair of networks in every trial.

The set of three LFP timeseries obtained from simulation together with its causal configuration are called *example*. We generated 1,000 examples, each with three timeseries of 6,000 timepoints, for each of the 25 causal configurations described in section 2.1. The whole set of examples defined a dataset that we call *NN dataset* from now on.

A Matlab implementation of the neural network model that generates the NN dataset is available at: https://github.com/FBK-NILab/causality_prediction_cortical_model.

### 2.3. MAR model and dataset

The multivariate autoregressive (MAR) dataset is composed of multiple examples. Each example comprises a multivariate timeseries X together with its causal configuration *A*, defined below. The multivariate timeseries $X=\left\{X\left(t\right),t=0,1,\ldots ,N-1\right\},X\left(t\right)\in {\mathbb{R}}^{M\times 1}$ is defined as the linear combination of two *M*-dimensional multivariate time series $X_{s}$ and $X_{n}$

(9) $$X=(1-\gamma )X_{s}+\gamma X_{n}$$

where $X_{s}$ carries the causal information (signal), $X_{n}$ represents an additive noise corruption and γ∈[0, 1] tunes the signal-to-noise ratio. Each time point of $X_{s}$ and $X_{n}$ is computed by following the stationary MAR model

(10) $$\begin{array}{l} X_{s}(t)=\sum_{\tau =1}^{p}A_{s}^{(\tau )⊤}X_{s}(t-\tau )+\varepsilon_{s}(t)\\ X_{n}(t)=\sum_{\tau =1}^{p}A_{n}^{(\tau )⊤}X_{n}(t-\tau )+\varepsilon_{n}(t) \end{array}$$

where *p* is the order of the MAR model and represents the maximal time lag. ε_*s*_(*t*) and ε_*n*_(*t*) are the innovation processes, defined as realizations from a diagonal *M*-dimensional standard normal distribution. $A_{s}^{\left(\tau \right)},A_{n}^{\left(\tau \right)}\in {\mathbb{R}}^{M\times M},\tau =1,\ldots ,p$ are the coefficient matrices modeling the influence of the signal values at time *t*−τ on the current signal values, i.e., at time *t*. The coefficient matrices $A_{s}^{\left(\tau \right)}$ defines the process of causal-informative data generation. They are computed by corrupting with uniform noise the non-zero elements of the *M*×*M* binary matrix *A*, called *causal configuration matrix*, where *a*_*ij*_ = 1 means signal *i* causes signal *j* and 0 otherwise. In essence, *A* represents the causal graph that defines the MAR model, as described in section 2.1. Differently, $A_{n}^{\left(\tau \right)}$ described the noisy part of the signals and they are obtained by randomly generating *p* diagonal matrices.

As for the NN dataset, in the experimental setup we chose *M* = 3, i.e., X=(X,Y,Z), *p* = 10 and *N* = 6, 000 and generated a dataset of examples. Each example consisted in three timeseries of 6,000 timepoints each, generated by the MAR model of order 10, together with the related causal configuration *A*. For each of the 25 possible causal configuration matrices described in section 2.1, we generated 1000 examples. From now on, we refer to this dataset as the *MAR dataset*.

A Python implementation of the MAR model that generates the MAR dataset ia available at: https://github.com/FBK-NILab/causality_prediction_cortical_model.

### 2.4. Geweke index of causality

Consider a system of three stationary stochastic processes *X*_*t*_, *Y*_*t*_, and *Z*_*t*_, under the assumption of multivariate autoregressive model. The traditional pair-wise conditional approach to causal inference examines whether *Y* has a direct influence on *X* given the presence of *Z* by decomposing

(11) $$X_{t}=\sum_{i=1}^{\infty }a_{xx,i}X_{t-i}+\sum_{i=1}^{\infty }a_{xy,i}Y_{t-i}+\sum_{i=1}^{\infty }a_{xz,i}Z_{t-i}+\varepsilon_{x,t}.$$

An alternative reduced representation of *X*, which assumes that *Y* has no influence, is instead

(12) $$X_{t}=\sum_{i=1}^{\infty }{a^{\prime }}_{xx,i}X_{t-i}+\sum_{i=1}^{\infty }{a^{\prime }}_{xz,i}Z_{t-i}+{\varepsilon^{\prime }}_{x,t}.$$

The *Geweke index of causality in time domain*, *F*_*Y*→*X*|*Z*_, estimates which of the two regressions (11) and (12) better models the process *X*_*t*_ by computing the log-ratio of the residual variances:

(13) $$F_{Y\to X|Z}=\ln\frac{{\Sigma^{\prime }}_{xx}}{\Sigma_{xx}}$$

where $\Sigma_{xx}^{\prime }=\text{var}\left(\varepsilon_{xx}^{\prime }\right)$ and Σ_*xx*_ = var(ε_*xx*_) are the residual variances of the MAR models (Equations 11 and 12), respectively. Equation (13) is interpreted as the variation in prediction error when the past of *Y* is included in the regression.

Frequently, *F*_*Y*→*X*|*Z*_ is considered during the process of causal inference. The common practice is to look at *F*_*Y*→*X*|*Z*_ as the test statistic of a log-likelihood ratio test. In particular, under the null hypothesis of no causality, i.e., *H*_0_:*a*_*xy, i*_ = 0, ∀*i*, the Geweke measure has an asymptotic χ^2^ distribution up to a scaling factor which depends on the sample size and with degree of freedom equal to the difference in the number of parameters between Equations (11) and (12). Under the alternative hypothesis, the scaled test statistic has an asymptotic non-central χ^2^ distribution, with non-centrality parameter that corresponds to the scaled casual measure. See Barnett and Seth (2014) for further details.

### 2.5. Supervised causal detection between timeseries

Recently, the machine learning literature has started to address the problem of causality (see Schölkopf et al., 2013). In Benozzo et al. (2017), we presented the first method to detect causality among timeseries, based on supervised learning and tested on the MAR model. Here we summarize that method, which we propose to use in conjunction with the NN model of section 2.2, instead of the MAR model.

Given three timeseries {**x**, **y**, **z**}, the set of all possible causal interactions considered in this study comprises 25 configurations (see Figure 1). In a supervised learning setting, we aim at creating a classifier that, given {**x**, **y**, **z**}, accurately predicts their causal configuration, among the 25 alternatives. Such classifier can be obtained in two steps: first, by designing a convenient feature space where to represent the set of timeseries in terms of potentially useful causal quantities. Second, by training a classification algorithm on a large dataset of known examples, where one example is a set of timeseries represented in the feature space together with its causal configuration. In sections 2.2 and 2.3, we described how to generate two alternative large datasets of examples, the first based on a novel neural network model and the second on the traditional MAR model.

The feature space that we proposed in Benozzo et al. (2017) is based on the idea of precedence and predictability, typical of the Granger causality framework. For each single timeseries, e.g., **x**, we quantify its degree of predictability according to different causality scenarios, as illustrated in Table 1. A causality scenario is a possible causal relationship that can explain the observed **x**. For example, in scenario 4 of Table 1, (**x**, **y**) are jointly *causes* of the *effect* **x**. We quantify the plausibility of each scenario with a measure of how good the fit of a plain multivariate linear regression of the effect is, at each timestep, from the past of the given causes. Following the previous example, for each *t*, we regress the value of *x*_*t*_ from past values of **x** and **y**, i.e., (*x*_*t*−*p*_, …, *x*_*t*−1_, *y*_*t*−*p*_, …, *y*_*t*−1_), and measure the goodness of fit with a score, such as the mean squared error. The lower the mean squared error, the higher the likelihood of such causality scenario at that timepoint. Notice that, in Benozzo et al. (2017), the past of the causes is taken into account till *p* time steps before *t*. There, the parameter *p* was introduced because the generative model considered there was the MAR model of order *p*. Such parameter, used to build the feature space, can be set to a value specific for the model that generated the timseries. In our case, for the parameter *p*, we can use one value for the MAR dataset and a different value for the NN dataset (see section 3.2).

**Table 1:** Given **x** as effect, we report the seven possible causality scenarios that can be obtained from the three timeseries **x**, **y**, and **z**, when considered as causes.

	**Causes**	**Effect**
1	**x**	**x**
2	**y**	**x**
3	**z**	**x**
4	**x**, **y**	**x**
5	**x**, **z**	**x**
6	**y**, **z**	**x**
7	**x**, **y**, **z**	**x**

The average score that is obtained over all timesteps represents the likelihood of such scenario for the whole multivariate timeseries. Such number is one feature of the vector representation of the multivariate timeseries, i.e., it is one of the dimensions of the feature space designed in Benozzo et al. (2017). In this way, the 7 causality scenarios of Table 1 generates 21 feature values, i.e., 7 for **x**, 7 for **y** and 7 for **z**. We adopted three scores: the mean squared error (mse), the coefficient of determination *R*^2^ and the Granger Causality index^1^ (gci), thus increasing the total number of feature values^2^ to 48. To this set of features we applied standard feature engineering techniques, i.e., we created additional features meant to help the classification system to learn non-linear effects in the data. As in Benozzo et al. (2017), we adopted the square root, second and third power of all previous features, i.e., 48 × 3 additional features, as well as all pairwise products of the features within each score, i.e., 210 + 210 + 15 = 435 additional features. The final vector representation of the initial multivariate timeseries consisted of 627 features. To conclude, each example of both the NN dataset and the MAR dataset was transformed into this feature space. In these newly transformed datasets, an example consists of a vector labeled with one of the 25 causality configurations that generated the corresponding set of timeseries.

The second step of the supervised method for causality detection is based on training a classification algorithm, such as logistic regression, see for example Bishop (2013), on the obtained dataset. In case of successful training, the resulting classifier is then ready to accurately predict which of the 25 causal configurations a new set of timeseries belongs to. Of course, such new set of timeseries will have to be represented in the feature space described above in order to be presented to the classifier. In section 3 we will show the practical application of this process to the simulated datasets.

## 3. Experiments and results

In this section we describe the two main experiments that we conducted in order to support the claims of the paper, together with some additional findings. The first experiment consists in characterizing the proposed neural network model with traditional metrics, in order to provide meaningful sanity checks for the simulations produced by the model. The second experiment compares the proposed supervised classification method for causality detection with the Granger Causality Analysis (GCA, see Barnett and Seth, 2014), commonly used to detect causality among timeseries from neural recordings. The ability of the two methods to detect the causal configurations among timeseries are compared on the data generated by the neural network model. In order to show the ability of the proposed supervised method to adapt to the given generative model, we also train the classifier on the MAR dataset and show that, in this case, it behaves similarly to GCA.

### 3.1. Traditional analyses

We conducted two traditional analyses on the sets of timeseries of the NN dataset, in order to show that the proposed neural network model generates timeseries in agreement with the causality architecture of the neural circuits. As described in section 2.2, the activity of each circuit in the neural network model was represented as the LFP generated by that circuit. Using the LFP representation of network activity is meaningful for several reasons: first, LFPs are a commonly-applied measure of the mass activity of neural circuits that samples neurons in regions roughly of the size of the circuits modeled here (see Buzsáki et al., 2012; Einevoll et al., 2013). Second, LFP captures a wide range of frequencies of neural activity (Buzsáki et al., 2012; Einevoll et al., 2013) that have been implicated in the coding of cortical information and in transmission of this information to other cortical areas (see Singer, 1999; Fries, 2005; Womelsdorf et al., 2007; Belitski et al., 2008; Ray and Maunsell, 2011; Jia et al., 2013; van Kerkoerle et al., 2014; Besserve et al., 2015; van Vugt et al., 2018).

The first analysis is based on observing the cross-correlation between two timeseries in presence and absence of causality link. The second analysis is based on quantifying the Geweke index of causality, that we described in section 2.4. On average, on all timeseries, we expect an higher degree of cross-correlation between timeseries in case there is a causal link between them. In the same way, the value of the Geweke index should show the presence/absence of causal link.

#### 3.1.1. Cross-correlation

**Figure 3:**
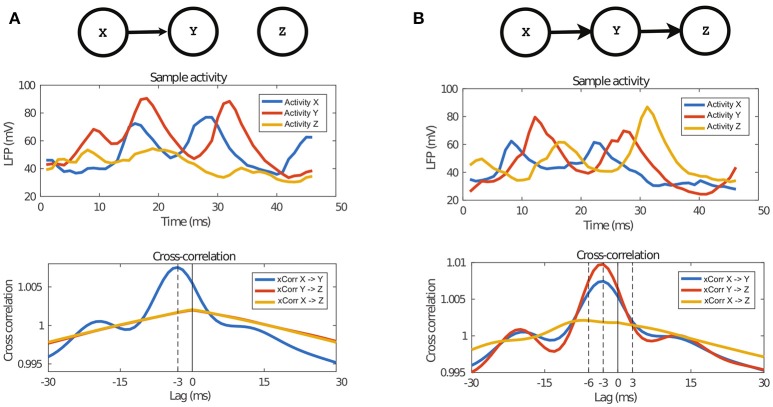
Properties of the data generated by the NN model. The top panel describes the architecture of the two causal configuration graphs, the middle one shows a sample of the activity of those networks and finally the last panel show the average cross-correlation between the activity. **(A)** In case of the univariate connection, it can be observed that activity of *Y* follows activity of *X*, which is also confirmed by the cross-correlation peak at the time equal to the transfer delay, 3 ms. **(B)** Also in the case of bivariate transfer, it can be observed that the activities follow each other, according to the causal configuration graph, which is also confirmed by the cross-correlation graph. The peak of cross-correlation between *X* and *Z* is at 6 ms because of the double delay between them.

We computed the cross-correlation between pairs of timeseries under some causality scenario, specifically the univariate and bivariate cases. In Figure 3, we show the two causal graphs describing those scenarios, the related sample of LFPs activity and the averaged cross-correlation graph between pairs of timeseries.

In Figure 3A, we report the univariate case (*X*→*Y*) and in Figure 3B the bivariate case (*X*→*Y*→*Z*). In both cases, we observe a peak of cross-correlation between timeseries with causal link, peaking at −3 ms, which matches the latency of inter-network connection defined in the proposed neural network model (see section 2.2). The standard deviation of cross-correlation, not reported in Figure 3, is always lower than 0.05 for *X*→*Y* and always lower than 0.045 for *X*→*Z* and *Y*→*Z*. For *X*→*Y*, the cross-correlation at the peak is 1.007, while the corresponding one when there is no causal link is 1.001. Such difference of means is significant when tested with a *t*-test^3^. It is interesting to note that, in the bivariate case, i.e., *X*→*Y*→*Z*, there are significant peaks at −3 ms both for *X*→*Y* and *Y*→*Z*, but also a shallow peak for *X*→*Z* at −6 ms, indicating anecdotal evidence of the indirect causal link between *X* and *Z*.

#### 3.1.2. Geweke index

**Figure 4:**
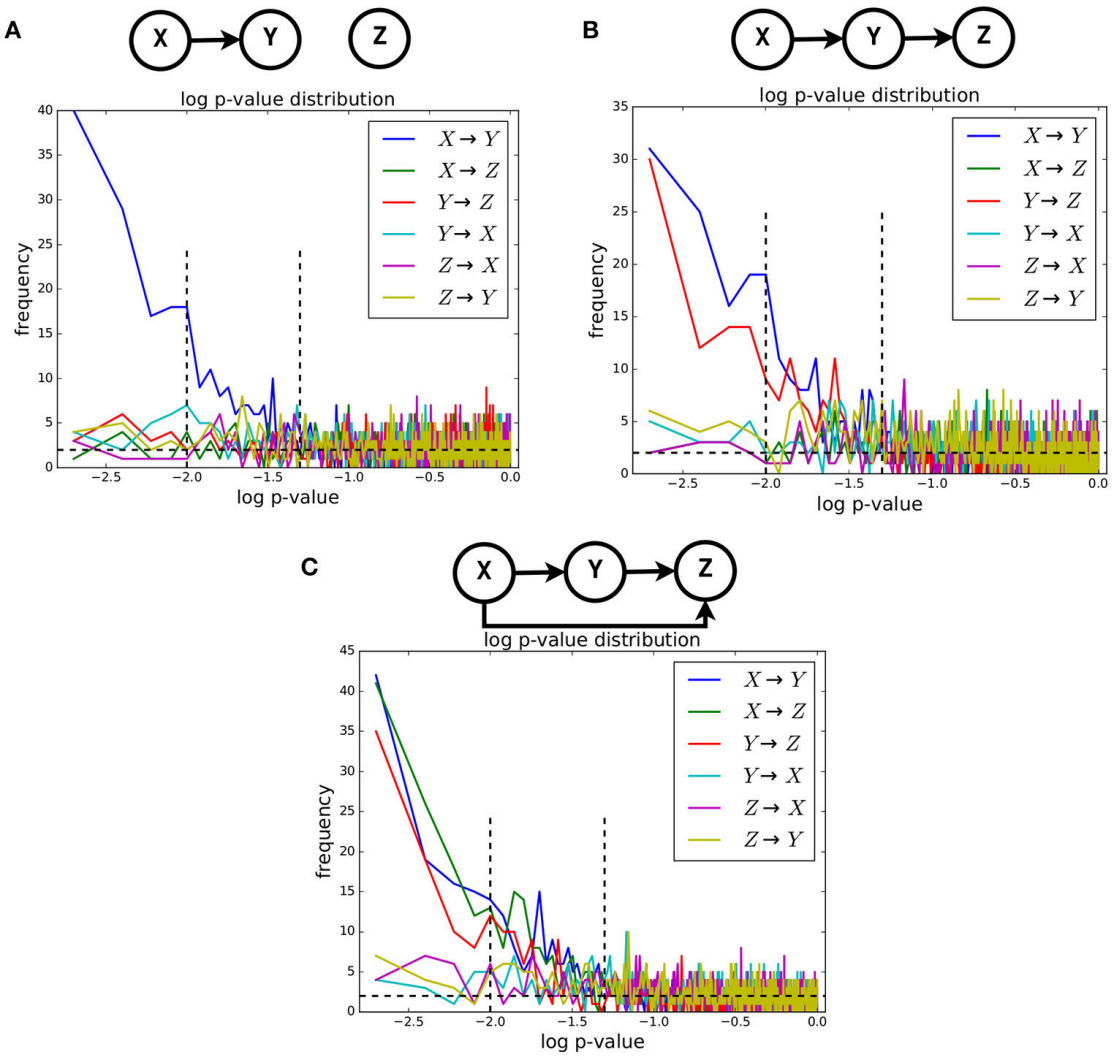
The approximate distributions, in log-scale, of the *p*-value of the Geweke test statistic between pairs of timeseries. Three causality configurations were considered: **(A)**, bivariate **(B)**, and trivariate **(C)**. In case of causal link between two timeseries, the distribution of *p*-value is expected to be non-uniform.

We computed the Geweke index of causality, introduced in section 2.4, between pairs of timeseries for all examples of the NN dataset belonging to three causality scenarios: univariate, bivariate and trivariate. For each scenario, we obtained the *p*-value distribution of such index for all possible ordered pairs, i.e., *X*→*Y*, *X*→*Z*, *Y*→*X*, *Y*→*Z*, *Z*→*X*, and *Z*→*Y* (see Figure 4). Given two timeseries in one example, the *p*-value of their Geweke index was computed considering the asymptotic χ^2^ distribution mentioned in section 2.4. The actual value was obtained with the Granger Causality Analysis (GCA) Toolbox (see Barnett and Seth, 2014). For each causality scenario, the approximate distributions of the *p*-value were obtained by computing such values over the related 1,000 examples available in the dataset.

It is known that, when the null hypothesis is true, the distribution of the *p*-value is uniform^4^. When the null hypothesis is false, such distribution is different from the uniform one. In our case, when the null hypothesis of *no causal link* between two timeseries is *true*, the corresponding approximate distribution of the Geweke index is expected to be uniform, and non-uniform when such hypothesis is false. In Figure 4, we show the approximate distributions of the *log*(*p*)-value computed on pairs of timeseries in case of presence and absence of such causal link, for the univariate (Figure 4A), bivariate (Figure 4B), and trivariate (Figure 4C) cases. It is clearly visible that such distributions are non-uniform only when the causal link exists. Expectedly, in that case they exhibit a highly peaked shape for low-*p*-values.

### 3.2. Supervised causality detection

In contrast to the previous experiment, which characterized causality on average on all dataset, in this experiment we tested the ability of the supervised causality method, described in section 2.5, to detect the full pattern of causal configurations from a *single* multivariate timeseries, under the assumption of being generated by the neural network model proposed in section 2.2. In order to show the ability of the supervised method to adapt to the assumed generative model of the data, we tested both the case when the training of the underlying classifier was conducted on the NN dataset and on the MAR dataset. Additionally, we compared the results with the commonly adopted Granger Causality Analysis method (GCA, see Barnett and Seth, 2014), based on the Geweke index of causality of section 2.4, that is used as state-of-the-art benchmark when detecting causality between neural recordings.

We transformed the NN dataset and MAR dataset, initially generated as described in sections 2.2 and 2.3, into datasets of class-labeled vectors using the feature space representation described in section 2.5. For the MAR dataset, for the features obtained through regression of the signal *p*-timesteps before, we selected *p* = 10 according to the value used to generate the dataset. For the NN dataset, we set *p* = 3 according to the inter-network connection parameter, τ_*L*−*int*_, defined in section 2.2 and the results in Figure 3.

**Figure 5:**
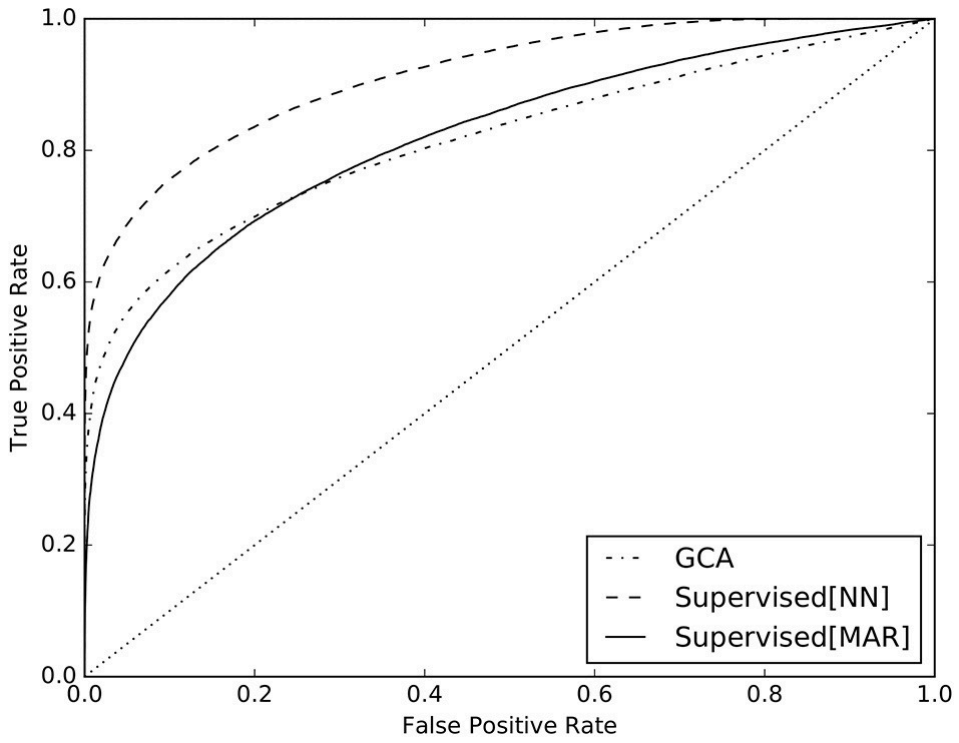
ROC curves for the predictions of the supervised method trained on the NN dataset (solid line), on the MAR dataset (dashed-dotted line), and of GCA (dashed line).

With a 5-fold cross-validation scheme, we split five times the NN dataset in train set and test set. The first one was used to train the Logistic Regression classifier of the supervised method and the second one to test its predictions. At the same time, we used GCA on the the NN dataset to compare the ability of detection of the two methods^5^. In analogy to the results that we presented in Benozzo et al. (2016), in Figure 5 we present the receiver operating characteristic (ROC) curve^6^ of the obtained predictions for the supervised method (solid line) and GCA (dashed line). Additionally, in order to show the ability of the supervised method to adapt to the generative model of the data and its dependence on it, we trained the Logistic Regression algorithm on the MAR dataset and tested it again on the NN dataset. The ROC curve of its predictions is reported in Figure 5 (dashed-dotted line). To conclude, we report in Table 2 the AUC values of the ROC curves presented in Figure 5, together with their bootstrap-estimated confidence interval.

**Table 2:** AUC-values of the ROC curves presented in Figure 5, i.e., for GCA, for the supervised method trained on the NN dataset and trained on the MAR dataset.

	**GCA**	**Supervised (NN)**	**Supervised (MAR)**
AUC	0.8160 ± 0.0008	**0.9139** ± 0.0005	0.8178 ± 0.0007

*The highest AUC score is indicated in bold face*.

Python code of the proposed method for this experiment is available at: https://github.com/FBK-NILab/causality_prediction_cortical_model.

## 4. Discussion and conclusions

In this section, we discuss the results obtained in the experiments presented in section 3 with respect to our claims. Additionally, we present current limitations of the proposed method and future perspectives. At the end of the section, we conclude the article describing this work.

### 4.1. Implications of our main findings

The neural network model proposed in section 2.2, that combines three interacting neuronal circuits, was built by extending the neural circuit model introduced in Mazzoni et al. (2008) to the multiple-area case. One important finding of this work is to show that the proposed model is sound and produces timeseries of LFPs in agreement with the causality architecture designed in the network. This is a useful finding in the context of our work, because it is known that there is not always a one-to-one correspondence between the structure of the anatomical connections and the resulting causal functional interactions. In Figure 3, we presented the cross-correlation between the timeseries from the NN dataset, averaged over all examples. There and in section 3.1.1, we showed that the average cross-correlation has a clear peak at −3 ms, when there is a causal link between the respective circuits, e.g., *X*→*Y* of Figure 3A. This value perfectly matches the designed inter-network latency of post-synaptic currents, τ_*L*−*int*_ = 3 ms, introduced in section 2.2. Such peak is present both in case of univariate and bivariate causal configurations. Conversely, when there was no causal link between circuits, the peak was absent, e.g., *X*→*Z* in Figure 3A. Additionally, in case of bivariate causal configuration, e.g., *X*→*Y*→*Z*, see Figure 3B, the analysis of cross-correlation revealed a shallow peak at −6 ms between the timeseries *X* and *Z*, giving anecdotal evidence that, on average, *Z* required 6 ms to show the behavior caused by *X*. Indeed, this peak is at twice the latency τ_*L*−*int*_ = 3 ms, i.e., once for *X*→*Y*, plus once for *Y*→*Z*, in agreement with the designed causality among the three neuronal circuits.

The results reported in Figure 3 are reassuring but not sufficient to confirm causality, since correlation does not mean causation. The results presented in Figure 4 are meant to analyze more in detail the timeseries generated by the proposed neural network model. There, we report the approximate distribution of the p-value of the Geweke index of causality between pairs of timeseries, across the NN dataset for univariate, bivariate and trivariate causal configurations. By definition, under the null hypothesis of no causal link between two timeseries, such distribution is uniform. Figure 4 clearly shows that, at an aggregate level over all examples for a given causal configuration, the presence of causality between two timeseries creates strongly non-uniform distributions, in all cases. As a consequence, we can state that LFPs generated by the proposed neural network model showed the expected patterns of Granger causality. This is a notable result, because the Granger assumption was not directly used in defining the proposed neural network model (see section 2.2).

The results discussed so far analyzed causality between timeseries at an aggregate level, i.e., as a summary over all examples belonging to one causal configuration. However, in practical cases, we are faced with the more difficult task of estimating the causal configuration of a single multivariate timeseries, usually measured during experiments. The GCA method of Barnett and Seth (2014) and the proposed supervised method, are meant to predict the causal configuration of single multivariate timeseries. The ROC curves in Figure 5 and the AUC values in Table 2 clearly show that the proposed supervised method, when trained on examples from the NN dataset, vastly outperforms GCA in detecting causality, increasing AUC from 0.81 to 0.91 (see curve and values labeled as Supervised[NN]). This finding is due to the fact that the supervised method is designed to adapt to the generative model through the simulated dataset, specifically in the training phase of the classifier. Conversely, GCA is designed on the assumptions of the MAR model, which substantially differs from the NN model. Such difference, prevents GCA to be accurate on NN data.

A further element in support to the claimed ability of the supervised method to adapt to the generative model, is the result regarding the ROC curve and the AUC value, labeled as Supervised[MAR] in Figure 5 and Table 2. In that case, the supervised method was trained on examples from the MAR dataset and tested on examples from the NN dataset. We clearly see that the results are equivalent to those of GCA: the two ROC curves, labeled as Supervised[MAR] and GCA, mostly overlap and the respective AUC values are almost equal. In essence, the supervised method is similar to GCA when trained on MAR data and much superior to GCA when trained on NN data.

### 4.2. Limitations and possible extensions in future work

A limitation of the work presented here is that we considered only networks of up to three nodes. An interesting question regards how this approach would scale-up when considering more nodes. As noted in section 2.1, the number of possible connectivity patterns between the nodes grows super-exponentially (see Equation 1). This is an intrinsic problem when studying causality between multiple interacting units. Even though such problem is not specific of the proposed methods, it has a clear impact on them, both on simulations and on the prediction step, as follows.

Considering solely the parameters of individual networks, the simulation time would scale exponentially with the number of neurons and linearly with both the number of connections between neurons within the same network and the number of connections between neurons belonging to different networks. In the case of the integrate and fire networks considered here, collecting a large number of samples from these simulations would be practical, on a modern multiprocessor workstation, only up to 5 nodes.

Computational limitations of the prediction step has been discussed in Benozzo et al. (2016) and we briefly summarize them here. The super-exponential growth in causality configurations, i.e., in the number of classes of the classifier, inherently hinders the possibility to build an accurate classifier when the number of nodes is greater than 4. This can be mitigated by adopting a different classification scheme, where each non-diagonal entry of the causality matrix is delegated to a different classifier, whose number grows only quadratically with the number of nodes. In Benozzo et al. (2016), such solution, named *cell-based classification*, has shown equivalent results with the one described in this paper, at the cost of requiring the training of multiple classifiers. A second scalability issue lies in the construction of the feature space: the number of causality scenarios described in Table 1 grows exponentially with the number of nodes, without considering the feature engineering step described in section 2.5. The only solution that we foresee is to approximate the causality detection problem by reducing the number of causality scenarios to a small subset of them, for example considering no more than the trivariate case. Furthermore, a simple way to avoid the substantial increase in the number of features due to feature engineering, is to completely avoid feature engineering and to directly rely on non-linear classification algorithms. Clearly, all these solutions come at a cost and trade-offs. In future, we plan to conduct experiments on all these aspects.

A further limitation of the current study is the observational assumption, typical of many frameworks of causality like the Granger one. Under such assumption only observed nodes are considered when modeling causality. Clearly, not considering non-observed nodes reduces the accuracy of detecting the causality pattern. In future, we plan to consider other causality frameworks, that can model interventional data and deal with confounding variables, possibly leading to a more accurate detection.

Despite the issues to be solved to scale-up these methods to a larger number of nodes, being able to practically and robustly evaluate causality between a handful of nodes could be already of great utility in current systems neuroscience. For example, it would allow to study information processing within several important stations of the visual system or of other sensory modalities (Wang and Burkhalter, 2007; DiCarlo et al., 2012; Tafazoli et al., 2017).

### 4.3. Conclusions

In this work we proposed a novel extension of an established neural network model, aimed at designing interactions among neuronal circuits. We proved that timeseries of LFPs generated from such model do follow the univariate, bivariate or trivariate causality structure designed between the circuits, as defined by Granger causality measures.

Unfortunately, there are no available solutions to invert this model, in order to infer the pattern of causality between timeseries from experimental data. For this reason, as a second main contribution, we present a supervised causality detection method that we previously introduced and applied to MAR models. Here, we show that the proposed method can very effectively adapt to the proposed neural network model and can estimate the pattern of causality between timeseries, vastly better than the commonly adopted GCA. For example, as shown in Figure 5, when setting the rate of false positives (FPR) to 10%, the supervised method correctly detects causality in 3 over 4 cases (TPR = 75%), while GCA only in less than 2 over 3 cases (TPR = 62%).

In future, as discussed above, we plan to investigate the detection of causality in multivariate timeseries from systems with more than three neuronal circuits. There, the challenge is to effectively address the super-exponential growth in the number of causality configurations with the number of circuits. A second interesting direction of research is improving the accuracy of the classification step, for example by using non-linear classification algorithms instead of the adopted (linear) Logistic Regression classifier.

## Author contributions

EO, JB, DB, SP and PA conceived the proposed methods and designed the experiments. EO wrote the manuscript. JB, DB, SP, PA contributed to it. JB and DB conducted the experiments.

### Conflict of interest statement

The authors declare that the research was conducted in the absence of any commercial or financial relationships that could be construed as a potential conflict of interest.

## Footnotes

^1^See GrangerAnlayzer in NiTime, http://nipy.org/nitime

^2^21 features for mse, 21 for *R*^2^ and 6 for gci, because it operates on pairs of timeseries.

^3^We used the Welch's t-test for unequal variances. Since each causal configuration comprises 1,000 examples, the *p*-value is 0.0024, which can be considered significant even with modern, more stringent, standards (Benjamin et al., 2017).

^4^This is a classical result. Given a test statistic *T*, with a certain null distribution *F*(*T*), its *p*-value *P* = *F*(*T*) has the following probability distribution: *Pr*(*P*<*p*) = *Pr*(*F*^−1^(*P*) < *F*^−1^(*p*)) = *Pr*(*T*<*t*) = *p*. Which means that *P* has uniform distribution.

^5^Notice that GCA is an unsupervised method which, differently from classification algorithms, does not require a train set. A fair comparison between GCA and the proposed supervised method requires the same test set. This is the reason why we used a cross-validation scheme for the supervised method.

^6^GCA predicts a score for each entry of the causal configuration matrix *A*, which is the likelihood of *a*_*ij*_ = 1. We set a threshold on the score and, for each example in the test set, we counted FPs and TPs. By averaging over all the test set, we obtained a point (FPR,TPR) and, by varying the threshold, the whole ROC curve. Supervised[MAR] and Supervised[NN] jointly predict the likelihood of the 25 possible causal configuration matrices. We set a threshold on the likelihood value and combined the matrices over threshold into one, such that *a*_*ij*_ = 1 if one of them has *a*_*ij*_ = 1, and 0 otherwise. Then we computed FPs, TPs, FPR, TPR and the ROC curve like for GCA.
